# Body Weight Variation Patterns as Predictors of Cognitive Decline over a 5 Year Follow-Up among Community-Dwelling Elderly (MAPT Study)

**DOI:** 10.3390/nu11061371

**Published:** 2019-06-18

**Authors:** Kelly Virecoulon Giudici, Sophie Guyonnet, Yves Rolland, Bruno Vellas, Philipe de Souto Barreto, Fati Nourhashemi, on behalf of the MAPT/DSA Group

**Affiliations:** 1 Gerontopole of Toulouse, Institute of Ageing, Toulouse University Hospital (CHU Toulouse), 31000 Toulouse, France; guyonnet.s@chu-toulouse.fr (S.G.); rolland.y@chu-toulouse.fr (Y.R.); vellas.b@chu-toulouse.fr (B.V.); philipebarreto81@yahoo.com.br (P.d.S.B.); nourhashemi.f@chu-toulouse.fr (F.N.); 2 UPS/Inserm UMR1027, University of Toulouse III, 31000 Toulouse, France

**Keywords:** cognition, weight loss, aging, hippocampal atrophy, elderly, Alzheimer’s disease

## Abstract

This study aimed to analyze associations between weight variation patterns and changes in cognitive function and hippocampal volume among non-demented, community-dwelling elderly. Sample was formed of 1394 adults >70 years (63.9% female), all volunteers from the Multidomain Alzheimer Preventive Trial (MAPT). Weight loss was defined as ≥5% of body weight decrease in the first year of follow-up; weight gain as ≥5% of weight increase; and stability if <5% weight variation. Cognition was examined by a Z-score combining four tests. Measures were assessed at baseline, 6, 12, 24, 36, 48, and 60 months of follow-up. Hippocampal volume was evaluated with magnetic resonance imaging in 349 subjects in the first year and at 36 months. Mixed models were performed. From the 1394 participants, 5.5% (*n* = 76) presented weight loss, and 9.0% (*n* = 125) presented weight gain. Cognitive Z-score decreased among all groups after 5 years, but decline was more pronounced among those who presented weight loss (adjusted between-group mean difference vs. stable: −0.24, 95%CI: −0.41 to −0.07; *p* = 0.006). After 3 years, hippocampal atrophy was observed among all groups, but no between-group differences were found. In conclusion, weight loss ≥5% in the first year predicted higher cognitive decline over a 5 year follow-up among community-dwelling elderly, independently of body mass index.

## 1. Introduction

Body composition is known to influence several chronic diseases and metabolic disturbances in all life stages. It is well established that obesity is a condition that increases the risk of cardiovascular diseases, type 2 diabetes, and many types of cancer [1]. Obesity in midlife has been shown to contribute to dementia [2,3], due to decreasing blood supply to the brain [4] and increasing adipocyte-secreted proteins and inflammatory cytokines that damage the brain’s white matter, leading to loss of cognitive and intellectual performance [5,6]. Further, insulin resistance and impaired glucose homeostasis are highly associated with obesity and are known to negatively affect cognition [7] through increasing β-amyloid levels in the brain [8]. On the other hand, later life elevated body mass index (BMI) seems to confer a lower risk of dementia [3,9]. In contrast, being underweight at older ages (including low muscle mass) has been related to impaired cognitive function [3,10,11,12,13].

While the relationship between nutritional status and cognition has been explored in literature and suggests that the predictive ability of BMI changes over a life time, the way body weight variation over a short period (such as one year) is able to influence cognition among elderly people has been less investigated. There is evidence that, at older ages, weight loss can be harmful and contribute to functional disability [14], cognitive impairment [9,15,16], and brain atrophy [17], which are known to associate with the development of Alzheimer’s disease [16,17,18].

We hypothesized that weight loss at older ages may associate with cognitive decline and with hippocampal atrophy (i.e., hippocampal volume loss), independently of BMI. Thus, the present study aimed to analyze how cognitive function (measured through a composite Z-score combining four tests) varied over a 5 year follow-up according to body weight variation patterns among non-demented, community-dwelling elderly people. As a secondary objective, hippocampal volume changes according to weight variation patterns were also evaluated over a 3 year follow-up in a subsample of participants.

## 2. Methods

This study is a secondary analysis with an observational approach, using data from the Multidomain Alzheimer Preventive Trial (MAPT). Briefly, MAPT was created to investigate the efficacy of omega-3 polyunsaturated fatty acid supplementation and a multidomain intervention including nutritional counseling, physical activity advice, and cognitive training in preventing cognitive decline over a 3 year follow-up, as thoroughly described elsewhere [19,20]. Participants were then followed for an additional period of 2 years without any intervention.

### 2.1. Study Population

Included subjects were non-demented (with a Mini-Mental State Examination—MMSE score ≥ 24) [21], community-dwelling women and men aged ≥70 years with slow gait speed (<0.8 m/s, measured by a 4 meter usual walking test), or limitation in executing at least one instrumental daily activity, or spontaneous memory complaints. Recruitment started in May 2008 and ended in February 2011. Follow-up ended in April 2016.

MAPT included 1679 volunteers attending 13 health centers in France. Participants with missing body weight measured at baseline or at 12 months were not included in the present study, leading to a final sample of 1394 subjects. All participants signed an informed consent. The MAPT study protocol was approved by the Advisory Committee for the Protection of Persons participating in Biomedical Research of the Toulouse University Hospital, and was authorized by the French Health Authority. The protocol (NCT00672685) can be found on a public access clinical trial database (www.clinicaltrials.gov).

### 2.2. Confounders

Sociodemographic information considered as potential confounders consisted of sex, age, and education (no diploma or primary school certificate, secondary education, high school diploma, university level). Other potential confounders considered in the study were the body mass index (calculated as weight in kg divided by height^2^ in m^2^), MAPT’s group assignment (multidomain intervention based on cognitive training, physical activity counseling, and nutritional counseling with omega-3 supplementation; multidomain intervention with placebo; omega-3 supplementation; and placebo alone) [19], incident cancer and ability in performing activities of daily living, which was measured by the Alzheimer’s Disease Cooperative Study Activities of Daily Living-Prevention Instrument (ADCS ADL-PI)—an instrument with a maximum score of 45, in which higher is better [22].

### 2.3. Weight Variation Patterns

Body weight was measured by a nurse according to standard procedures [23]. Weight loss was defined as ≥5.0% of body weight decrease in the first year of follow-up (between baseline and the 1 year visit); accordingly, weight gain was defined as ≥5.0% of body weight increase, and stability as <5.0% of weight variation. This cutoff was established due to consistency in the literature showing that reducing ≥5.0% of body weight over a period of 6 months to 1 year is associated with higher morbidity and mortality among elderly subjects [24,25,26].

### 2.4. Outcomes: Cognitive Score and Hippocampal Volume

Following the same criteria used in the main MAPT analysis, cognition was evaluated by a composite Z-score based on the sum of Z-scores from four cognitive tests divided by four: free and total recall of the Free and Cued Selective Reminding Test (FCSRT), the ten orientation items of the MMSE, Digit Symbol Substitution Test (DSST), and Category Naming Test (CNT). In order to avoid learning effects, two different word lists for the Free and Cued Selective Reminding Test were alternately used in participants’ visits. Measurements were performed by trained neuropsychologists, physicians, and nurses. Variables were assessed at baseline, 6, 12, 24, 36, 48, and 60 months of follow-up.

Hippocampal volume was determined by magnetic resonance imaging (MRI), and measures were generated using an automated procedure (SACHA Software) [27], as previously described elsewhere [19]. This exam was performed in a subsample of 503 participants from 9 health centers at the first year of the study and repeated in 379 subjects at 36 months of follow-up. The first scan was performed between January 2010 and September 2011, and the second measure was taken between January 2012 and September 2014. Hippocampal volume measures at both moments were available for 349 subjects, which were included in the present study.

### 2.5. Statistical Analysis

Means and standard deviation or frequencies and percentages were used to describe the studied sample. The normality of the distribution of each continuous variable was assessed with the Kolmogorov–Smirnov test, and logarithmic transformation was used if needed. Analysis of variance (ANOVA) was used to compare means according to weight variation patterns (weight loss, stable, or weight gain). Categorical variables were compared using the chi-square test. Linear mixed effects models were performed to explore the variation in the cognitive score (dependent variable) over the 5 year follow-up according to weight variation patterns (independent variable). Time was used as a continuous variable, and, firstly, unadjusted linear mixed models with a random effect at the participant level were performed with the following fixed effects: weight variation patterns, time, time^2^, time^3^, and interaction between patterns and each time term. Adjusted linear mixed models taking into account potential confounders were then performed, including sex, age, educational level, BMI, ADCS ADL-PI score, incident cancer, MAPT intervention groups, interaction between weight variation patterns and MAPT groups, and the interaction between each time term and these variables. Similarly, mixed models were performed to explore the variation in hippocampal volume (dependent variable) over a 3 year follow-up according to weight variation patterns (independent variable), but in these models time^2^ and time^3^ were not retained, due to not reaching statistical significance.

In order to reduce the risk of reverse causality between body weight variation and cognitive decline, sensitivity analysis excluding participants with baseline composite cognitive Z-score values below the 10th percentile was performed. This cutoff was chosen due to its showing significantly greater sensitivity and equivalent specificity for screening dementia in a population-based study [28]. Statistical Analysis Software (SAS) version 9.4 (Cary, NC, USA) was used for all analyses, and results were considered statistically significant if *p* < 0.05.

## 3. Results

### 3.1. Characterization of the Sample

A total of 1394 participants were evaluated in this analysis, 63.9% female, mean age 75.2 years (standard deviation—SD = 4.3). Baseline characteristics according to weight variation patterns are presented in Table 1. Weight loss ≥5% over 1 year was observed among 5.5% (*n* = 76) of the sample, while weight gain ≥5% over 1 year was observed among 9.0% of participants (*n* = 125). A higher proportion of women was observed among the weight gain group. Participants in the weight loss group presented higher BMI and lower composite cognitive Z-score, lower DSST score, and lower free and total recall scores of the FCSRT at baseline, compared to those who remained stable over time. This group also was older, and presented higher BMI and lower free and total recall scores of the FCSRT at baseline compared to those in the weight gain group. Participants in the weight gain group were shorter and presented lower body weight and lower BMI at baseline compared to the stable group.

### 3.2. Changes in Cognitive Z-score According to Body Weight Variation Patterns

At the end of the 5 year follow-up, cognitive Z-score decreased among all groups of weight variation. However, within-group declines were more pronounced among participants in the weight loss group (−0.50, 95% CI: −0.67 to −0.33; *p* < 0.0001). Comparison in cognitive Z-score changes between groups revealed significant differences between weight loss pattern and the stable group (mean between-group difference: −0.27, 95% CI: −0.44 to −0.10; *p* = 0.002), and results persisted after adjustments for potential confounders (−0.24, 95% CI: −0.41 to −0.07; *p* = 0.006). Meanwhile, no significant differences were found between those in the weight gain pattern and the stable group (Table 2).

Sensitivity analysis retaining only participants with baseline cognitive Z-score above the 10th percentile (*n* = 1251) provided similar results. Cognitive Z-score decreased among all weight variation patterns, and more pronounced declines were observed among participants in the weight loss group (−0.45, 95% CI: −0.61 to −0.29; *p* < 0.0001). Between-group differences were found comparing the weight loss pattern and the stable group (mean between-group difference: −0.21, 95% CI: −0.38 to −0.05; *p* = 0.013), and results persisted after adjustments for potential confounders (−0.19, 95% CI: −0.35 to −0.02; *p* = 0.028) (Table 3).

### 3.3. Changes in Hippocampus Volume According to Body Weight Variation Patterns

Analysis of a subsample of participants with MRI data (*n* = 349) are detailed in Table 4, showing changes in hippocampal volume according to weight variation patterns. Hippocampal atrophy was observed among all groups of weight variation after 36 months. No differences were observed between weight variation patterns in both unadjusted and adjusted models.

## 4. Discussion

The present study tested if body weight variation patterns over the first year of the study would be able to independently associate with future changes in cognition up to 5 years, among a sample of non-demented community-dwelling elderly people. Cognitive function, measured by a composite score combining four tests, declined among all groups over time, but weight loss ≥5% over one year was associated with higher decline in cognitive score compared to weight maintenance, independently of BMI and other potential confounders. Analysis with a subsample who had MRI information revealed that all three body weight variation patterns presented significant hippocampal volume decrease after 36 months, however, for this variable, no between-group differences were found.

The relationship between body weight variation and cognition among older adults has shown mixed evidence in literature, accentuating the complex interaction between energy metabolism and brain function. In accordance with our findings, a good amount of evidence points towards a prejudicial effect of weight loss during the aging process on cognitive impairment in different populations [9,13,15,16,17,29]. Indeed, the relationship between weight loss and dementia has also been reinforced by a study with data from eight low and middle-income countries evaluating 16,538 adults ≥65 years old [30], and by a recent study examining 67,219 individuals aged 60–79 years [31]. On the other hand, improved cognitive function after weight loss has also been observed among elderly people [32,33,34]. In this sense, it is worth discussing how weight loss may differently affect cognition when being a consequence of metabolic disturbances rather than a desired and controlled situation.

Unintentional weight loss is frequent at older life stages, and is often associated with increased frailty, nutritional unbalance, and functional decline [14,35], leading to higher mortality among elderly people [14,25,36]. An accentuated, unplanned decrease in body weight involves bone mineral density (BMD) loss [37], and, more importantly, lean mass loss, which is markedly associated with changes in the immune system (comprising both a decline in immune function with aging and a state of chronic inflammation) [38]. Besides the interaction between frailty, sarcopenia, and immunosenescence as a triad favoring cognitive impairment, understanding mechanisms which may trigger or fasten such conditions is highly relevant. Causes of unintentional weight loss among adults and elderly have been explored, and depression, cancer, and gastrointestinal tract disorders stand out as the major ones [24,39]. Thus, despite a percentage of unknown causes in the studies (varying from 3 to 36%), in most part of the cases, unintentional weight loss—comprising lean mass decrease—is clearly an undesired consequence of chronic diseases and/or its medications’ side effects, which can lead to anorexia (and consequent insufficient food intake), nutrient malabsorption, and/or increased energy expenditure [39]. Such associated muscle loss may reduce strength, which in turn would possibly favor cognitive impairment through reducing functional abilities and physical performance [40,41].

On the other hand, intentional weight loss has been associated with distinct findings on cognitive function, even if also including decreases in lean mass. A review study including ten randomized controlled trials found that most interventions targeting weight loss among obese older adults (≥65 years) reported a loss of lean body mass and BMD with weight loss [42]. Paradoxically, muscle quality and physical function were improved, which could be possibly explained by the positive effect of physical activity in attenuating muscle loss and reducing inflammatory molecules such as tumor necrosis factor alpha (TNF-α) and interleukin-6 (IL-6) [42]. Other studies have also shown improved cognitive function after weight loss interventions [32,33,34,43,44] or bariatric surgery among obese individuals [45,46]. In this sense, it is presumable that, among elderly individuals with excessive body weight, well-monitored weight loss associated with exercising may be safe and beneficial to health (including to cognitive function), while unintentional weight loss (as a proxy of metabolic disturbances) would be a clear predictor of cognitive impairment.

Our study also evaluated hippocampal volume as a secondary outcome of cognitive impairment in a subsample of participants, however, no differences according to weight variation patterns were observed. One possible explanation for the lack of difference may be the shorter time range of follow-up (3 years, while cognitive Z-score was evaluated for 5 years). Given that cognitive Z-score was affected by weight loss but hippocampal volume was not, it is also possible that our participants’ cognitive decline may not necessarily fit in a pathway leading to Alzheimer’s disease, but rather in a context of cognitive frailty, a heterogeneous clinical manifestation which may represent a precursor of neurodegenerative processes [47]. On the other hand, other studies have shown unintentional weight loss to be associated with hippocampal atrophy among elderly subjects [48,49]. Contrasting to these results, intentional weight loss after caloric restriction has been shown to improve memory and to associate with gray matter volume increase in the right hippocampus in older obese women [50]. Findings of this study were specific for transient negative energy balance and were not detected after subsequent weight maintenance, inferring that beneficial effects of caloric restriction on brain structure and function were due to weight loss itself rather than an overall reduced weight [50]. This group of evidence reinforces the distinction in metabolic responses between intentional and unintentional weight loss in older ages. Besides the anthropometric measures, cognitive tests, and imaging explored in our study, future perspectives in the field include further research on biological mechanisms in order to settle the association between weight changes and cognitive decline. For example, metabolic pathways that might affect this relationship in the context of aging include those involving sirtuins. In experimental studies, this group of enzymes have shown an ability to act in adipocytes inhibiting adipogenesis [51] and to protect against cellular oxidative stress [52] (which is known to contribute with cognitive impairment [53]). In addition, overexpression of sirtuin-related genes has been associated with longevity in lower organisms [54]. The potential role of sirtuins in mediating the association between weight variation and cognition deserves further investigation.

Some strengths of the present study should be noted. Its longitudinal design enabled investigation of long-term associations in a large sample of older adults. The long duration of follow-up covered several assessments of cognitive tests, allowing close following of the outcome over time in analyses that included multiple potential confounders. A subanalysis assessing hippocampal volume changes, measured by MRI, provided a second outcome related with Alzheimer’s disease development. In addition, sensitivity analysis excluding participants with baseline cognitive Z-score below the 10th percentile was performed to reduce the risk of reverse causality, and ratified our findings. On the other hand, some limitations can be mentioned. This study represents a secondary analysis with a population participating in a randomized trial which involved intervention (not aiming for weight changes) until the third year of follow-up. In order to reduce bias, the intervention group assignment was considered in the adjusted models, as was the interaction between weight variation patterns and the group assignment. The MRI was restricted to a subsample of participants and limited to two measurements. Despite adding several important potential confounders in our analysis (such as cancer incidence and BMI), other parameters such as genetic variabilities were not considered, and may be explored in future studies. Lastly, we observed a particularly high educational level of volunteers in our sample, which suggests a high level of cognitive reserve, so caution is needed when comparing the findings with different populations.

## 5. Conclusions

Our study found weight loss to be an independent predictor of higher cognitive decline after a 5 year follow-up among a sample of non-demented, community-dwelling French elderly people. Our findings highlight the importance of monitoring weight variation in the aging, and reinforce how practices targeting body composition changes among elderly patients must be based on multiple, rather than on isolated, aspects. Given the increase in life expectancy and the consequent raise in the number of older adults and in the prevalence of chronic diseases, fighting obesity in this population is of especial interest, but particularly challenging due to exacerbation of the age-related loss of skeletal muscle and bone over fat reduction (which can be attenuated, but not blocked, with physical activity [42]). A clinically global evaluation must be done in order to evaluate the potential risks and benefits of this prognosis, considering all aspects related to physical and mental health. In this sense, better understanding of how weight variation independently affects cognitive function is imperative. Future studies are needed in order to settle how body composition variation along with aging predicts cognitive decline early among elderly people, and to strengthen preventive strategies against Alzheimer’s disease and other dementias.

## Figures and Tables

**Table 1:** Baseline characteristics of the studied sample according to weight variation patterns (Multidomain Alzheimer Preventive Trial—MAPT study).

	Total *n* = 1394	Weight Variation Patterns		
		Weight Loss	Stable	Weight Gain
		*n* = 76	*n* = 1193	*n* = 125
	Mean ± SD *	Meean ± SD *	Meean ± SD *	Meean ± SD *
Female sex	890 (63.9%)	48 (63.2%)	747 (62.6%)	95 (76.0%) †
Age (years)	75.2 ± 4.3	75.9 ± 4.3 ^a^	75.2 ± 4.4	74.6 ± 4.0 ^a^
Education (*n* = 1375)				
No diploma or primary school certificate	298 (21.7%)	18 (23.7%)	260 (22.2%)	20 (16.0%)
Secondary education	468 (34.0%)	22 (29.0%)	400 (34.1%)	46 (36.8%)
High school diploma	204 (14.8%)	13 (17.1%)	178 (15.2%)	13 (10.4%)
University level	405 (29.5%)	23 (30.3%)	336 (28.6%)	46 (36.8%)
Weight (kg)	68.6 ± 12.9	70.9 ± 13.6 ^a^	68.9 ± 12.8 ^b^	64.4 ± 12.4 ^a,b^
Height (m)	1.62 ± 0.09	1.61 ± 0.09	1.62 ± 0.09 ^a^	1.60 ± 0.08 ^a^
Body mass index (kg/m^2^)	26.1 ± 4.0	27.3 ± 4.5 ^a,b^	26.2 ± 4.0 ^a,c^	25.0 ± 4.0 ^b,c^
Cognition				
Composite Z-score **	0.02 ± 0.66	−0.13 ± 0.70 ^a^	0.03 ± 0.65 ^a^	0.01 ± 0.66
Free and total recall of the Free and Cued Selective Reminding test score	73.1 ± 9.5	70.6 ± 11.6 ^a,b^	73.2 ± 9.2 ^a^	73.6 ± 10.1 ^b^
Ten Mini-Mental State Examination orientation items score	9.8 ± 0.5	9.8 ± 0.5	9.8 ± 0.5	9.8 ± 0.5
Digit Symbol Substitution Test score	38.1 ± 9.9	35.7 ± 9.6 ^a^	38.3 ± 9.9 ^a^	38.2 ± 9.7
Category Naming Test score	26.1 ± 7.4	25.7 ± 6.9	26.3 ± 7.4	25.1 ± 8.1

* except where indicated other; ** based on four cognitive tests (free and total recall of the Free and Cued Selective Reminding test, ten Mini-Mental State Examination orientation items, Digit Symbol Substitution Test and Category Naming Test); † *p* < 0.05; ^a,b,c^ same letters indicate difference between groups (*p* < 0.05).

**Table 2:** Mixed-effect linear regression analysis for variation in composite cognitive Z-score over a 5 year follow-up according to weight variation patterns among non-demented, community-dwelling elderly.

	Estimated Mean Within-Group 5 Year Change from Baseline * (95% CI); *p*-Value			Between-Group Differences in Cognitive Score after 5 Year Follow-Up (95% CI); *p*-Value			
	Weight Loss	Stable	Weight Gain	Weight Loss vs. Stable		Weight Gain vs. Stable	
				Unadjusted Model	Adjusted Model **	Unadjusted Model	Adjusted Model **
Composite cognitive score	−0.47 (−0.61, −0.33); <0.0001	−0.21 (−0.24, −0.18); <0.0001	−0.14 (−0.25, −0.04); 0.007	−0.26 (−0.40, −0.11); 0.001	−0.24 (−0.41, −0.07); 0.006	0.07 (−0.04, 0.18); 0.222	0.07 (−0.06, 0.19); 0.287

CI: confidence interval; * Negative values indicate cognitive score impairment; ** Model adjusted by age, sex, body mass index, educational level, MAPT intervention groups, Alzheimer’s Disease Cooperative Study Activities of Daily Living—Prevention Instrument (ADCS ADL-PI) score, interaction between weight patterns and MAPT groups, and time interactions.

**Table 3:** Mixed-effect linear regression analysis for variation in composite cognitive Z-score over a 5 year follow-up according to weight variation patterns among non-demented, community-dwelling elderly. Results in this table are restricted to participants with baseline cognitive Z-score higher than percentile 10 (n = 1251).

	Estimated Mean Within-Group 5 Year Change from Baseline * (95% CI); *p*-Value			Between-Group Differences in Cognitive Score after 5 Year Follow-Up (95% CI); *p*-Value			
	Weight Loss	Stable	Weight Gain	Weight Loss vs. Stable		Weight Gain vs. Stable	
				Unadjusted Model	Adjusted Model **	Unadjusted Model	Adjusted Model **
Composite cognitive score	−0.42 (−0.56, −0.29); <0.0001	−0.21 (−0.24, −0.18); <0.0001	−0.15 (−0.25, −0.05); 0.005	−0.21 (−0.36, −0.07); 0.003	−0.19 (−0.35, −0.02); 0.028	0.06 (−0.04, 0.17); 0.244	0.06 (−0.06, 0.19); 0.305

CI: confidence interval; * Negative values indicate cognitive score impairment; ** Model adjusted by age, sex, body mass index, educational level, MAPT intervention groups, Alzheimer’s Disease Cooperative Study Activities of Daily Living—Prevention Instrument (ADCS ADL-PI) score, interaction between weight patterns and MAPT groups, and time interactions.

**Table 4:** Hippocampus volume changes measured by magnetic resonance imaging (MRI) over a 3 year follow-up according to body weight variation patterns among non-demented, community-dwelling elderly people.

	Estimated Mean Within-Group 3 Year Change from Baseline * (95% CI); *p*-Value			Between-Group Differences in Hippocampal Volume after 3 Year Follow-Up (95% CI); *p*-Value			
	Weight Loss	Stable	Weight Gain	Weight Loss vs. Stable		Weight Gain vs. Stable	
				Unadjusted Model	Adjusted Model **	Unadjusted Model	Adjusted Model **
Hippocampal volume (cm^3^)	−0.14 (−0.21, −0.08); <0.0001	−0.12 (−0.14, −0.11); <0.0001	−0.10 (−0.14, −0.06); <0.0001	−0.02 (−0.09, 0.05); 0.536	0.01 (−0.06, 0.08); 0.788	0.02 (−0.02, 0.06); 0.379	0.00 (−0.05, 0.04); 0.912

CI: confidence interval; * Negative values indicate hippocampal atrophy; ** Adjusted by age, sex, body mass index, educational level, MAPT intervention groups, Alzheimer’s Disease Cooperative Study Activities of Daily Living—Prevention Instrument (ADCS ADL-PI) score, interaction between weight patterns and MAPT groups, and time interactions.

## MAPT/DSA Group

Principal investigator: Bruno Vellas (Toulouse); Coordination: Sophie Guyonnet; Project leader: Isabelle Carrié; CRA: Lauréane Brigitte; Investigators: Catherine Faisant, Françoise Lala, Julien Delrieu, Hélène Villars; Psychologists: Emeline Combrouze, Carole Badufle, Audrey Zueras; Methodology, statistical analysis and data management: Sandrine Andrieu, Christelle Cantet, Christophe Morin; Multidomain group: Gabor Abellan Van Kan, Charlotte Dupuy, Yves Rolland (physical and nutritional components), Céline Caillaud, Pierre-Jean Ousset (cognitive component), Françoise Lala (preventive consultation) (Toulouse). The cognitive component was designed in collaboration with Sherry Willis from the University of Seattle, and Sylvie Belleville, Brigitte Gilbert and Francine Fontaine from the University of Montreal. Co-Investigators in associated centres: Jean-François Dartigues, Isabelle Marcet, Fleur Delva, Alexandra Foubert, Sandrine Cerda (Bordeaux); Marie-Noëlle-Cuffi, Corinne Costes (Castres); Olivier Rouaud, Patrick Manckoundia, Valérie Quipourt, Sophie Marilier, Evelyne Franon (Dijon); Lawrence Bories, Marie-Laure Pader, Marie-France Basset, Bruno Lapoujade, Valérie Faure, Michael Li Yung Tong, Christine Malick-Loiseau, Evelyne Cazaban-Campistron (Foix); Françoise Desclaux, Colette Blatge (Lavaur); Thierry Dantoine, Cécile Laubarie-Mouret, Isabelle Saulnier, Jean-Pierre Clément, Marie-Agnès Picat, Laurence Bernard-Bourzeix, Stéphanie Willebois, Iléana Désormais, Noëlle Cardinaud (Limoges); Marc Bonnefoy, Pierre Livet, Pascale Rebaudet, Claire Gédéon, Catherine Burdet, Flavien Terracol (Lyon), Alain Pesce, Stéphanie Roth, Sylvie Chaillou, Sandrine Louchart (Monaco); Kristelle Sudres, Nicolas Lebrun, Nadège Barro-Belaygues (Montauban); Jacques Touchon, Karim Bennys, Audrey Gabelle, Aurélia Romano, Lynda Touati, Cécilia Marelli, Cécile Pays (Montpellier); Philippe Robert, Franck Le Duff, Claire Gervais, Sébastien Gonfrier (Nice); Yannick Gasnier and Serge Bordes, Danièle Begorre, Christian Carpuat, Khaled Khales, Jean-François Lefebvre, Samira Misbah El Idrissi, Pierre Skolil, Jean-Pierre Salles (Tarbes). MRI group: Carole Dufouil (Bordeaux), Stéphane Lehéricy, Marie Chupin, Jean-François Mangin, Ali Bouhayia (Paris); Michèle Allard (Bordeaux); Frédéric Ricolfi (Dijon); Dominique Dubois (Foix); Marie Paule Bonceour Martel (Limoges); François Cotton (Lyon); Alain Bonafé (Montpellier); Stéphane Chanalet (Nice); Françoise Hugon (Tarbes); Fabrice Bonneville, Christophe Cognard, François Chollet (Toulouse). PET scans group: Pierre Payoux, Thierry Voisin, Julien Delrieu, Sophie Peiffer, Anne Hitzel, (Toulouse); Michèle Allard (Bordeaux); Michel Zanca (Montpellier); Jacques Monteil (Limoges); Jacques Darcourt (Nice). Medico-economics group: Laurent Molinier, Hélène Derumeaux, Nadège Costa (Toulouse). Biological sample collection: Bertrand Perret, Claire Vinel, Sylvie Caspar-Bauguil (Toulouse). Safety management: Pascale Olivier-Abbal. DSA Group: Sandrine Andrieu, Christelle Cantet, Nicola Coley.
